# The Causal Association Between Obesity and Primary Open-Angle Glaucoma: A Two-Sample Mendelian Randomization Study

**DOI:** 10.3389/fgene.2022.835524

**Published:** 2022-04-25

**Authors:** Yi Lin, Xiaomin Zhu, Wangdu Luo, Bingcai Jiang, Qianyi Lin, Min Tang, Xiangji Li, Lin Xie

**Affiliations:** Department of Ophthalmology, The Third Affiliated Hospital of Chongqing Medical University, Chongqing, China

**Keywords:** primary open-angle glaucoma, body mass index, waist circumference, hip circumference, Mendelian randomization

## Abstract

The correlation between obesity and primary open-angle glaucoma (POAG) has not yet been fully established. The aim of this study was to investigate the causal relationship between obesity and POAG by a two-sample Mendelian randomization (MR) study. In this study, body mass index (BMI), an index to evaluate general obesity, and waist and hip circumference, indices to evaluate abdominal obesity, were selected as exposures in MR analysis. Single-nucleotide polymorphisms (SNPs) were chosen as instrumental variables (IVs). Summary data from genome-wide association studies (GWASs) based on a European ancestry by Locke et al., with regard to BMI, and Shungin et al., with regard to waist and hip circumference, were used. Genetic predictors of POAG were obtained from public GWAS summary data. To assess the causal effect of obesity on POAG, the inverse variance-weighted (IVW) method was used as the primary method, and other methods, such as MR–Egger, weighted median, simple mode, and weighted mode, were also used as complementary analyses. Finally, we performed Cochran’s Q statistic to assess heterogeneity, and sensitivity analysis was performed to evaluate the reliability and stability of the MR results. MR analysis showed that BMI has a positive effect on the risk of POAG, with 1 standard deviation (SD) increase in BMI; the risk of POAG increases by approximately 90.9% [OR = 1.909; 95% CI= (1.225, 2.975); *p* = 0.0042)] (analyzed by IVW); there were no heterogeneity and pleiotropy in the result; and waist circumference also had a positive effect on the risk of POAG [OR = 2.319; 95% CI= (1.071, 5.018); *p* = 0.033)] analyzed by weighted median. As hip circumference increases, with 1 SD increase in hip circumference, the risk of POAG increases by approximately 119% [OR = 2.199; 95% CI= (1.306, 3.703); *p* = 0.00305)] estimated by IVW, there were not heterogeneity and pleiotropy as for the result. Our study for the first time confirms that obesity might increase the risk of POAG using two-sample MR analysis. These results might provide guidance on the prevention and treatment of POAG.

## Introduction

Glaucoma is a leading cause of irreversible blindness worldwide and is mostly characterized by the progressive loss of retinal ganglion cells and their axons (Chang and Goldberg, 2012). Among them, primary open-angle glaucoma (POAG) is the most common type of glaucoma, affecting nearly 80 million people worldwide (Petriti et al., 2021). At present, its pathogenesis has not been fully elucidated, but it is well known that multifactorial events are involved in the development of POAG (Lichter, 2003; Jonas et al., 2017). In addition to pathologically high intraocular pressure (IOP) (A. Sommer et al., 1991), numerous risk factors, including genetic factors (Rong et al., 2016), systemic diseases (Wostyn et al., 2017), and environmental factors (B. Fan et al., 2004; Kountouras et al., 2008), have been found to play an role in the pathogenesis of POAG. As a complicated multifactor disease, clinical symptoms are usually not obvious at an early stage, and visual function is irreversibly damaged at onset (Gauthier & Liu, 2016). Therefore, it is of great significance to actively explore the risk factors for POAG development, which might promote earlier detection and reduce its incidence.

Obesity is a major global public health problem, and its incidence is on the rise in many countries (Y. Y. Wu et al., 2017). According to World Health Organization statistics, the prevalence of obesity (body mass index (BMI) ≥ 30 kg/m^2^) in the United States is expected to soar to 50.7% by 2030 (Finkelstein et al., 2012). Similarly, the rates of obesity in Europe and Asia have increased exponentially in the last decade (Whitton et al., 2017). Studies have shown that obesity has an extensive influence on human health and is a risk factor for diabetes, coronary heart disease, hypertension, and other diseases (Nagarajan et al., 2017). Currently, there is little research on the correlation between obesity and POAG, and it is still controversial whether obesity represents a risk factor for POAG. In a prospective cohort study in Korea, people with BMI ≥30 kg/m^2^ were more likely to develop POAG than those with BMI of 18.5–22.9 kg/m^2^ (Jung et al., 2019). Consistent with this result, a population-based study in the United States reported that obesity (BMI ≥30 kg/m^2^) was significantly correlated with the prevalence of glaucoma (Ko et al., 2016). In contrast, based on a population study in central India, a lower BMI was associated with a higher prevalence of glaucoma (Nangia et al., 2013). These controversial conclusions may be caused by different races, the existence of confounders, and biases in previous studies. Moreover, the genetic aspects might also have a role in the pathogenesis. Thus, the association between BMI and POAG remains unclear, and further research is warranted.

In traditional retrospective studies, confounders and biases cannot be eliminated, and the time sequence of exposure and outcome is often confused, which often leads to inconsistent and controversial results (Talari & Goyal, 2020). Randomized controlled trials (RCTs) are time-consuming and require a large number of staff and finances; moreover, it is often not ethical to conduct an RCT, so they are quite difficult to perform (Serra-Aracil et al., 2020). To address the aforementioned issues, Katan et al. (1986) introduced the concept of Mendelian randomization (MR) in 1986. MR is an epidemiological approach for investigating whether a causal effect exists between exposures and outcomes by using genetic variants as instrumental variables (IVs) (Yeung & Schooling, 2021). This approach uses genetic variants that depend on strongly associated single-nucleotide polymorphisms (SNPs) from genome-wide association studies (GWASs) and makes use of the random classification of alleles during gametogenesis, which is similar to randomized clinical trials (Qu et al., 2021). As a hot research tool in recent years, MR is not affected by common confounding factors, and the causal sequence is reasonable (Burgess et al., 2019; Skrivankova et al., 2021), which has unique advantages for judging causal inference between exposure factors and outcomes (Zhang et al., 2021).

The aim of this study was to explore the causal effect of obesity on POAG by two-sample Mendelian randomization. To the best of our knowledge, this was the first study to explore the causal effect of obesity on POAG by Mendelian randomization.

## Materials and Methods

### Study Design

**FIGURE 1:**
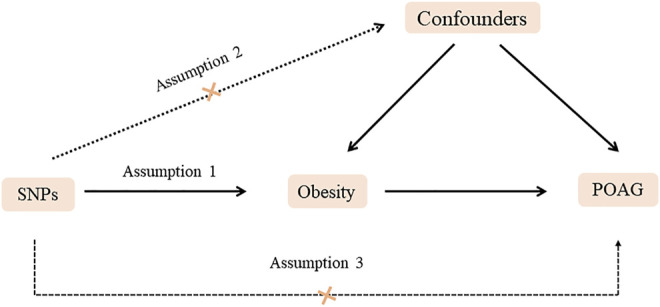
Diagram of two-sample Mendelian randomization analysis. Assumption 1: The SNPs should be associated with POAG. Assumption 2: The selected SNPs should be independent of confounders. Assumption 3: The SNPs should affect POAG only via obesity rather than via a direct correlation.

In the present work, obesity-associated indices, including BMI, waist circumference, and hip circumference, were chosen as exposures (Figure 1). We chose SNPs associated with exposure as IVs from GWAS summary datasets based on individuals of a European ancestry. The GWAS summary data published by Locke et al. (2015) with regard to BMI and Shungin et al. (2015) with regard to waist and hip circumference were adopted. Genetic predictors of POAG were obtained from public GWAS summary data (https://gwas.mrcieu.ac.uk/datasets), which were based on 1,824 POAG cases and 93,036 controls of individuals of a European descent. Consequently, SNPs were screened, and various statistical methods were used to assess the causal effects of obesity on the risk of POAG.

### Selection of Genetic Instrumental Variables

To evaluate the causal relationship between obesity (BMI, waist, circumference, and hip circumference) and POAG, SNPs were selected according to the following criteria: 1) SNPs highly associated with obesity and significant according to GWAS (*p* < 5.0 × 10^−8^); 2) SNPs independent of each other to avoid biases caused by linkage disequilibrium (*r* ^2^ < 0.0001/clumping, distance >5,000 kb; SNPs were extracted as IVs for further analysis); or 3) IVs could have an impact on the outcome only through exposure. In a two-sample MR, it is necessary to ensure that the affected allele of IVs in exposure and outcome between different databases corresponds to the same allele. Thus, allele frequency information was used to harmonize the data. The *F* statistics of each SNP is usually used to judge whether there were weak IVs. *F* statistics = *R* ^2^ (n-k-1)/k (1-R^2^), where *R* ^2^ is variance of exposure explained by selected instrumental variables, n is the sample size, and k is the number of instrumental variables (F. Wu et al., 2020). When the *F* statistics ≥10, it is generally considered that there were no biases caused by weak IVs. The details of the SNPs used in this study are provided in Supplementary File S1.

### Statistical Analysis

The standard inverse variance-weighted (IVW) method was applied to primarily analyze the causal relationships between obesity (BMI, waist, and hip circumference) and POAG (Brumpton et al., 2020; Park et al., 2021). This method calculated the Wald ratio of each SNP to assess the causal effects of each SNP on outcome, and finally, the inverse variances of SNPs were used as weights for meta-analysis to evaluate the combined causal effect. Furthermore, MR–Egger, weighted median, simple mode, and weighted mode were also used to evaluate the causal relationships between obesity (BMI, waist circumference, and hip circumference) and POAG. The MR–Egger method is used to assess whether genetic variants have pleiotropic effects on the outcome and also to obtain a consistent estimate of the causal effect (Burgess and Thompson, 2017). The weighted median is used to obtain valid estimates if at least 50% of the weight comes from valid variants (Luo et al., 2020). At last, sensitivity analysis was performed to evaluate the stability and reliability of MR results, including heterogeneity tested by Cochran’s Q test and I^2^ statistics (a *p*-value of <0.05 or an I^2^ value of >50% were regarded as significant heterogeneity), pleiotropy tested by using the MR–Egger intercept, and sensitivity by using the leave-one-out test.

All statistical analyses were performed in R software (version 3.6.1) with the R package “Two sample MR” (version 0.5.6) (Hemani G et al., 2018). P<0.05 was considered statistically significant. Moreover, the Benjamini–Hochberg method was used to obtain the adjusted *P* values. Since publicly available summary data were used in this study, the ethical approval was not required.

## Results

### Instrumental Variable Selection

To perform the MR analysis, significant and independent SNPs were extracted, and those with an *F-statistics* < 10 were excluded. Finally, a total of 31 BMI-related SNPs (the mean of *F*-statistics was 89.22), 33 waist circumference-related SNPs (the mean of *F*-statistics was 49.37), and 24 hip circumference-related SNPs (the mean of *F*-statistics was 68.78) were selected for two-sample MR analysis. All these SNPs are listed in Supplementary File S1.

### The Causal Relationship Between Body Mass Index and Primary Open-Angle Glaucoma

**FIGURE 2:**
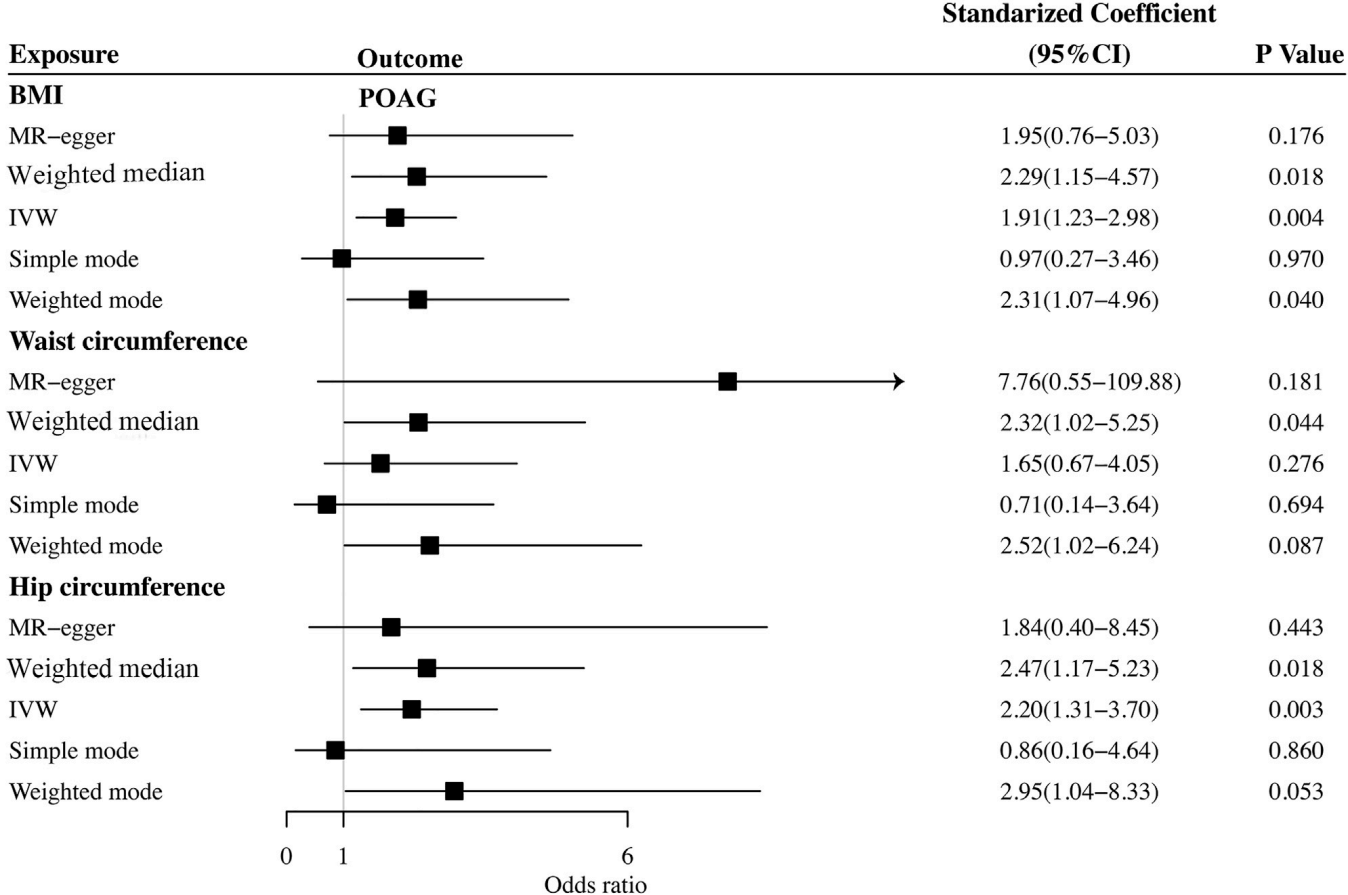
Summary of Mendelian randomization analysis results of the cause of obesity on POAG. BMI: body mass index; IVW: inverse variance weighted; POAG: primary open-angle glaucoma; MR: Mendelian randomization.

**FIGURE 3:**
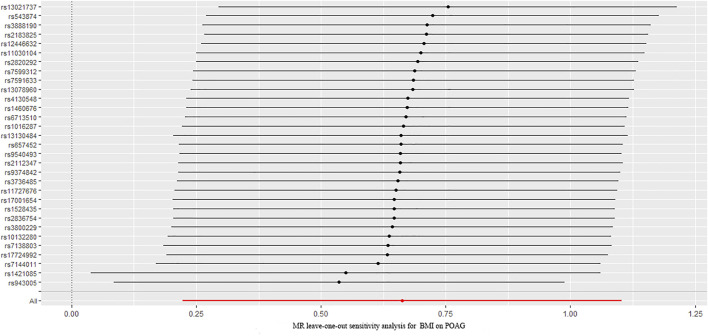
Leave-one-out analysis plots for BMI on the risk of POAG. BMI: body mass index; POAG: primary open-angle glaucoma; MR: Mendelian randomization.

The causal association between BMI and POAG assessed by two-sample MR analysis is summarized in Figure 2. As the results showed, BMI had a positive effect on the risk of POAG, with 1 SD increase in BMI, and the risk of POAG increased by approximately 90.9% [OR = 1.909; 95% CI= (1.225, 2.975); *p* = 0.0042; adjusted *P* = 0.0065)] according to the IVW method. The weighted median [OR = 2.294; 95% CI= (1.184, 4.442); *p* = 0.0138; adjusted *P* = 0.027)] also showed a similar causal relationship between BMI and POAG. The sensitivity analysis showed that there were no heterogeneities (Q-value = 24.20; *p* = 0.72; I^2^ = 11.96%) and no directional pleiotropies (MR–Egger intercept = −8.255 × 10^−4^; SE = 0.0151; *p* = 0.957). Moreover, the leave-one-out test showed that the MR results were not significantly affected by single SNP leave-out (Figure 3), indicating that the results were reliable and stable.

### The Causal Relationship Between Waist Circumference and Primary Open-Angle Glaucoma

**FIGURE 4:**
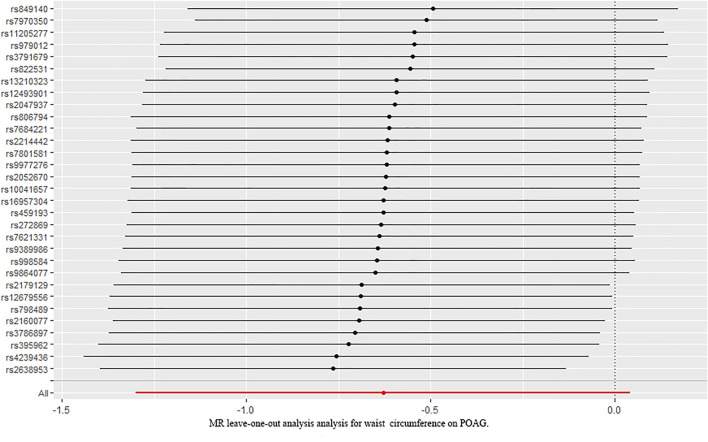
Leave-one-out analysis plots for waist circumference on the risk of POAG. POAG: primary open-angle glaucoma; MR: Mendelian randomization.

We further investigated the relationship between waist circumference and the risk of POAG by MR analysis (Figure 2). The results showed that there was a null causal effect of waist circumference on the risk of POAG [OR = 1.647; 95% CI= (0.671, 4.045); *p* = 0.276; adjusted *P*= 0.228)] analyzed by using the IVW method, while the weighted median showed a positive effect [OR = 2.319; 95% CI= (1.02, 5.25); *p* = 0.044; adjusted *P* = 0.0438)]. Since there was heterogeneity (Q-value = 15.89; *p* = 0.036) and a lack of directional pleiotropy (MR–Egger intercept = −0.0759; SE = 0.063; *p* = 0.271), according to Nazarzadeh et al. (2020), MR results analyzed by weighted median were adopted, indicating that the waist circumference has a positive effect on the risk of POAG. With 1 SD increase in hip circumference, the risk of POAG increased by approximately 132%. The leave-one-out test is illustrated in Figure 4.

### The Causal Relationship Between Hip Circumference and Primary Open-Angle Glaucoma

**FIGURE 5:**
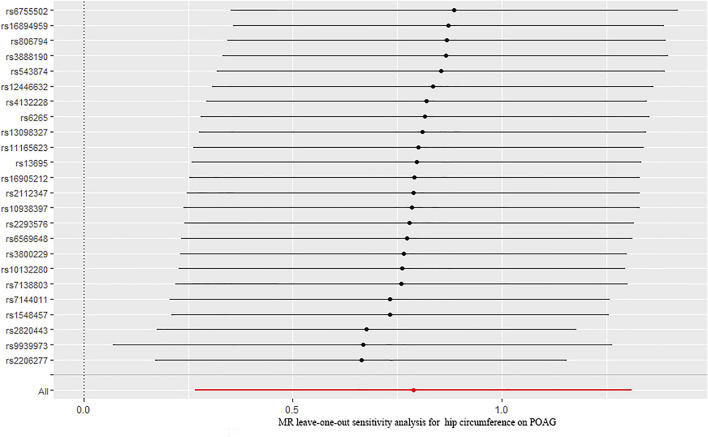
Leave-one-out analysis plots for hip circumference on the risk of POAG. POAG: primary open-angle glaucoma; MR: Mendelian randomization.

Finally, we explored the causal relationship between hip circumference and POAG. The MR results are shown in Figure 2. The results of IVW showed that hip circumference had a positive effect on the risk of POAG, with 1 SD increase in hip circumference, and the risk of POAG increased by approximately 119% [OR = 2.199; 95% CI= (1.306, 3.703); *p* = 0.00305; adjusted *P* = 0.00645)]. The weighted median results also confirmed this relationship [OR = 2.470; 95% CI= (1.167, 5.230); *p* = 0.018; adjusted *P* = 0.027)]. To assess the stability and reliability of these results, a sensitivity analysis was performed. The heterogeneity test showed that there were no heterogeneities (Q-value = 27.957; *p* = 0.217 and I^2^ = 31.5%), and the directional pleiotropies tested by using the MR–Egger intercept also showed that there were no pleiotropies (MR–Egger intercept = 6.417 × 10^−3^; SE = 0.0261; *p* = 0.808). Finally, the leave-one-out test indicated that the MR results were stable and were not significantly affected by single SNP leave-out (Figure 5).

## Discussion

Primary open-angle glaucoma is the most common type of glaucoma (Yuan et al., 2013). The early clinical manifestations are almost insidious, and visual function may be irreversibly damaged at onset (Song et al., 2018). Therefore, proactively identifying the risk factors for POAG has become the central point of POAG prevention. With the improvement of living conditions and changes in diet and lifestyles, overweight and obesity have become health issues that cannot be ignored and that pose a threat to the growth of adolescents globally (Reddy & Natarajan, 2011). However, whether obesity is a risk factor for POAG is still unclear; thus, it is important to clarify the relationship between obesity and POAG, which could provide guidance on the prevention and treatment of POAG in people with obesity.

Generally, obesity can be classified as general obesity, which is judged by BMI, and abdominal obesity, which is judged by waist circumference, hip circumference, or waist-to-hip ratio (WHR) (“Obesity: Preventing and Managing the Global Epidemic. Report of a WHO Consultation”; World Health Organization, 2000). It is widely acknowledged that obesity is a risk factor for many diseases, such as coronary atherosclerotic heart disease, hypertension, and diabetes (I. Sommer et al., 2020). To date, there are limited studies with regard to the relationship between obesity and POAG. The study performed by Wise et al. (2011) with 416,171 person-years of follow-up showed that BMI, waist circumference, and waist-to-hip ratio were significantly associated with POAG. Many other studies have also confirmed this conclusion. According to the available data, high intraocular pressure is a clear risk factor for glaucoma. Mori et al. (2000), who studied the relationship between obesity and intraocular pressure by cross-sectional and longitudinal analyses in Japan, showed that with the increase in BMI, the intraocular pressure also increased significantly. However, conversely, a study performed by Na et al. (2020) showed that being underweight was significantly related to an increased risk of POAG, with approximately 9.8 and 27.8% in individuals with and without diabetes, respectively. The causal relationship between obesity and POAG is still controversial and might be attributed to the differences in the selected cohort, different definitions or categories of diseases, or differences in follow-up periods. Thus, the correlation between obesity and POAG has not yet been fully established, and many previous studies were retrospective studies in which confounders could not be eliminated, so more studies should be performed, and much evidence should be collected to clarify this relationship.

In traditional observational epidemiological studies, confounders often interfere with the results, which makes the explanation of etiology unreliable. Mendelian randomization is an epidemiological research design and data analysis based on Mendelian independent distribution law, which is an unbiased estimation of causal effects (Shiose and Kawase, 1986; Smith and Ebrahim, 2003; Howe et al., 2020). In addition, the MR model of aggregated data takes GWAS based on a large sample as the dataset. Compared with models with small samples based on individual data, the acceptance of large samples greatly improves the inspection performance (Q. Fan et al., 2017; Lawlor et al., 2008). In this study, regardless of whether the choice of IVs was related to exposure or POAG as the outcome, the data used were from published large-scale GWAS data, and the results were more accurate, reliable, and convincing. In the present work, to comprehensively assess the causal effect of obesity on POAG, BMI, waist circumference, and hip circumference were selected as exposures to perform MR analysis. Our study demonstrated that BMI, waist circumference, and hip circumference have positive effects on the risk of POAG {[OR = 1.940; 95% CI= (1.249, 3.013); *p* = 0.00317)], [OR = 2.319; 95% CI=(1.071, 5.018); *p* = 0.033)], and [OR = 2.199; 95% CI=(1.306, 3.703); *p* = 0.00305)], respectively}. The causal relationships between BMI, hip circumference, and POAG were primarily analyzed by using the IVW method, and the sensitivity analysis revealed that there were no heterogeneities or pleiotropies. However, the causal association between waist circumference and POAG assessed by IVW suggested a null result. Since there was pleiotropy presence, the results analyzed by weighted median were adopted according to a previous study and showed a positive effect on POAG. This might be attributed to the database used in this study, and this result should be interpreted cautiously.

At present, there are some hypotheses about the mechanisms of how obesity increases the risk of POAG. On the one hand, too much adipose tissue might increase the pressure of the scleral vein and blood viscosity, improve the outflow resistance in the scleral vein, and cause a decrease in intraocular blood perfusion (Bulpitt et al., 1975). On the other hand, endocrine disorders in obese patients could lead to an increase in androgen, making the oxidative stress level in the body significantly higher than that in normal people; this, in turn, increases the possibility of oxidative damage in the outflow system of aqueous humor, such as trabecular meshwork, and blocks the outflow channel of aqueous humor, which significantly increases the risk of POAG (Oh et al., n. d.; Zarei et al., 2017).

Our study has some strengths: 1) We first assessed the causal relationship between obesity and POAG by MR analysis, in which confounders and biases could be largely eliminated. 2) BMI, waist circumference, and hip circumference were chosen as exposures to perform two-sample MR, which could comprehensively evaluate these causal effects. There were also some limitations in this study: 1) The two-sample MR analysis was based on a European ancestry, and this relationship might change in individuals of other ancestries; a two-sample MR analysis of individuals of at least one other ancestry should also be performed. 2) This study, which uses only summary statistics for MR analysis, could make only a preliminary judgment on the causal relationship between obesity and POAG, and the specific mechanisms of how obesity increases the risk of POAG still need further research.

In conclusion, our principal findings revealed that genetically predicted BMI, waist circumference, and hip circumference were related to the risk of POAG. To our knowledge, the present study performed a two-sample MR study for the first time to investigate the causal effect of obesity on POAG. To the best of our knowledge, this was the first study to investigate the causal effect of obesity on POAG, and our results further confirmed that obesity could increase the risk of POAG, which might provide guidance on the prevention and treatment of POAG.

## Data Availability

The original contributions presented in the study are included in the article/Supplementary Material; further inquiries can be directed to the corresponding author.

## References

[B1] Brumpton B. Sanderson E. Heilbron K. Hartwig F. P. Harrison S. Vie G. Å. (2020). Avoiding Dynastic, Assortative Mating, and Population Stratification Biases in Mendelian Randomization through Within-Family Analyses. Nat. Commun. 11 (1), 3519. 10.1038/s41467-020-17117-4 32665587 PMC7360778

[B2] Bulpitt C. J. Hodes C. Everitt M. G. (1975). Intraocular Pressure and Systemic Blood Pressure in the Elderly. Br. J. Ophthalmol. 59 (12), 717–720. 10.1136/bjo.59.12.717 1218183 PMC1017441

[B3] Burgess S. Davey Smith G. Davies N. M. Dudbridge F. Gill D. Glymour M. M. (2019). Guidelines for Performing Mendelian Randomization Investigations. Wellcome Open Res. 4, 186. 10.12688/wellcomeopenres.15555.2 32760811 PMC7384151

[B4] Burgess S. Thompson S. G. (2017). Interpreting Findings from Mendelian Randomization Using the MR-Egger Method. Eur. J. Epidemiol. 32 (5), 377–389. 10.1007/s10654-017-0255-x 28527048 PMC5506233

[B5] Chang E. E. Goldberg J. L. (2012). Glaucoma 2.0: Neuroprotection, Neuroregeneration, Neuroenhancement. Ophthalmology 119 (5), 979–986. 10.1016/j.ophtha.2011.11.003 22349567 PMC3343191

[B6] Davey Smith G. Ebrahim S. (2003). ‘Mendelian Randomization': Can Genetic Epidemiology Contribute to Understanding Environmental Determinants of Disease?*. Int. J. Epidemiol. 32 (1), 1–22. 10.1093/ije/dyg070 12689998

[B7] Fan B. J. Leung Y. F. Wang N. Lam S. C. Liu Y. Tam O. S. (2004). Genetic and Environmental Risk Factors for Primary Open-Angle Glaucoma. Chin. Med. J. (Engl) 117 (5), 706–710. 10.3760/cma.j.issn.0366-6999.2004.05.116 15161538

[B8] Fan Q. Maranville J. C. Fritsche L. Sim X. Cheung C. M. G. Chen L. J. (2017). HDL-cholesterol Levels and Risk of Age-Related Macular Degeneration: a Multiethnic Genetic Study Using Mendelian Randomization. Int. J. Epidemiol. 46 (6), 1891–1902. 10.1093/ije/dyx189 29025108 PMC5837540

[B9] Finkelstein E. A. Khavjou O. A. Thompson H. Trogdon J. G. Pan L. Sherry B. (2012). Obesity and Severe Obesity Forecasts through 2030. Am. J. Prev. Med. 42 (6), 563–570. 10.1016/j.amepre.2011.10.026 22608371

[B10] Gauthier A. C. Liu J. (2016). Neurodegeneration and Neuroprotection in Glaucoma. Yale J. Biol. Med. 89 (1), 73–79. 27505018 PMC4797839

[B11] Howe L. D. Kanayalal R. Harrison S. Beaumont R. N. Davies A. R. Frayling T. M. (2020). Effects of Body Mass Index on Relationship Status, Social Contact and Socio-Economic Position: Mendelian Randomization and Within-Sibling Study in UK Biobank. Int. J. Epidemiol. 49 (4), 1173–1184. 10.1093/ije/dyz240 31800047 PMC7750981

[B12] Jonas J. B. Aung T. Bourne R. R. Bron A. M. Ritch R. Panda-Jonas S. (2017). Glaucoma. The Lancet 390 (10108), 2183–2193. 10.1016/S0140-6736(17)31469-1 28577860

[B13] Jung Y. Han K. Park H.-Y. L. Lee S. H. Park C. K. (2020). Metabolic Health, Obesity, and the Risk of Developing Open-Angle Glaucoma: Metabolically Healthy Obese Patients versus Metabolically Unhealthy but Normal Weight Patients. Diabetes Metab. J. 44, 414–425. 10.4093/dmj.2019.0048 31950773 PMC7332336

[B14] Katan M. (1986). Apoupoprotein E Isoforms, Serum Cholesterol, and Cancer. The Lancet 327 (8479), 507–508. 10.1016/S0140-6736(86)92972-7 2869248

[B15] Ko F. Boland M. V. Gupta P. Gadkaree S. K. Vitale S. Guallar E. (2016). Diabetes, Triglyceride Levels, and Other Risk Factors for Glaucoma in the National Health and Nutrition Examination Survey 2005-2008. Invest. Ophthalmol. Vis. Sci. 57 (4), 2152–2157. 10.1167/iovs.15-18373 27111561 PMC4849858

[B16] Kountouras J. Zavos C. Grigoriadis N. Deretzi G. Katsinelos P. Tzilves D. (2008). *Helicobacter pylori* Infection as an Environmental Familial Clustering Risk Factor for Primary Open-Angle Glaucoma. Clin. Exp. Ophthalmol. 36 (3), 296–297. 10.1111/j.1442-9071.2008.01729.x 18412607

[B17] Lawlor D. A. Harbord R. M. Sterne J. A. C. Timpson N. Davey Smith G. (2008). Mendelian Randomization: Using Genes as Instruments for Making Causal Inferences in Epidemiology. Statist. Med. 27 (8), 1133–1163. 10.1002/sim.3034 17886233

[B18] Lichter P. R. (2003). Glaucoma Clinical Trials and What They Mean for Our Patients. Am. J. Ophthalmol. 136 (1), 136–145. 10.1016/s0002-9394(03)00143-0 12834681

[B19] Locke A. E. Kahali B. Berndt S. I. Justice A. E. Pers T. H. Day F. R. (2015). Genetic Studies of Body Mass Index Yield New Insights for Obesity Biology. Nature 518 (7538), 197–206. 10.1038/nature14177 25673413 PMC4382211

[B20] Luo S. Au Yeung S. L. Zuber V. Burgess S. Schooling C. M. (2020). Impact of Genetically Predicted Red Blood Cell Traits on Venous Thromboembolism: Multivariable Mendelian Randomization Study Using UK Biobank. Jaha 9 (14), e016771. 10.1161/JAHA.120.016771 32635790 PMC7660720

[B21] Mori K. Ando F. Nomura H. Sato Y. Shimokata H. (2000). Relationship Between Intraocular Pressure and Obesity in Japan. Int. J. Epidemiol. 29 (4), 661–666. 10.1093/ije/29.4.661 10922342

[B22] Na K.-S. Kim J.-H. Paik J.-S. Cho W.-K. Ha M. Park Y.-G. (2020). Underweight Increases the Risk of Primary Open-Angle Glaucoma in Diabetes Patients. Medicine 99 (10), e19285. 10.1097/MD.0000000000019285 32150063 PMC7478655

[B23] Nagarajan R. Carpenter C. L. Lee C. C. Michael N. Sarma M. K. Souza R. (2017). Assessment of Lipid and Metabolite Changes in Obese Calf Muscle Using Multi-Echo Echo-Planar Correlated Spectroscopic Imaging. Sci. Rep. 7 (1), 17338. 10.1038/s41598-017-17529-1 29229948 PMC5725420

[B24] Nangia V. Jonas J. B. Matin A. Bhojwani K. Sinha A. Kulkarni M. (2013). Prevalence and Associated Factors of Glaucoma in Rural Central India. The Central India Eye and Medical Study. PLoS One 8 (9), e76434. 10.1371/journal.pone.0076434 24098790 PMC3787001

[B25] Nazarzadeh M. Pinho-Gomes A.-C. Bidel Z. Dehghan A. Canoy D. Hassaine A. (2020). Plasma Lipids and Risk of Aortic Valve Stenosis: A Mendelian Randomization Study. Eur. Heart J. 41 (40), 3913–3920. 10.1093/eurheartj/ehaa070 32076698 PMC7654932

[B26] Oh S. W. Lee S. Park C. Kim D. J. (2005). Elevated Intraocular Pressure Is Associated with Insulin Resistance and Metabolic Syndrome. Diabetes Metab. Res. Rev. 21 (5), 434–440. 10.1002/dmrr.529 15651065

[B27] Park S. Lee S. Kim Y. Cho S. Kim K. Kim Y. C. (2021). A Mendelian Randomization Study Found Causal Linkage Between Telomere Attrition and Chronic Kidney Disease. Kidney Int. 100 (5), 1063–1070. 10.1016/j.kint.2021.06.041 34339747

[B28] Petriti B. Williams P. A. Lascaratos G. Chau K.-Y. Garway-Heath D. F. (2021). Neuroprotection in Glaucoma: NAD+/NADH Redox State as a Potential Biomarker and Therapeutic Target. Cells 10 (6), 1402. 10.3390/cells10061402 34198948 PMC8226607

[B29] Qu Z. Yang F. Hong J. Wang W. Li S. Jiang G. (2021). Causal Relationship of Serum Nutritional Factors with Osteoarthritis: A Mendelian Randomization Study. Rheumatology (United Kingdom) 60 (5), 2383–2390. 10.1093/rheumatology/keaa622 33167034

[B30] Reddy M. A. Natarajan R. (2011). Epigenetic Mechanisms in Diabetic Vascular Complications. Cardiovasc. Res. 90 (3), 421–429. 10.1093/cvr/cvr024 21266525 PMC3096305

[B31] Rong S. S. Tang F. Y. Chu W. K. Ma L. Yam J. C. S. Tang S. M. (2016). Genetic Associations of Primary Angle-Closure Disease. Ophthalmology 123 (6), 1211–1221. 10.1016/j.ophtha.2015.12.027 26854036

[B32] Serra-Aracil X. Pascua-Sol M. Badia-Closa J. Navarro-Soto S. Navarro Soto S. Sánchez Santos R. (2020). How to Start and Develop a Multicenter, Prospective, Randomized, Controlled Trial. Cirugía Española (English Edition), 98 (3), 119–126. 10.1016/j.ciresp.2019.11.012 31932028

[B33] Shiose Y. Kawase Y. (1986). A New Approach to Stratified Normal Intraocular Pressure in a General Population. Am. J. Ophthalmol. 101 (6), 714–721. 10.1016/0002-9394(86)90776-2 3717257

[B34] Shungin D. Winkler T. W. Winkler T. W. Croteau-Chonka D. C. Ferreira T. Locke A. E. (2015). New Genetic Loci Link Adipose and Insulin Biology to Body Fat Distribution. Nature 518 (7538), 187–196. 10.1038/nature14132 25673412 PMC4338562

[B35] Skrivankova V. W. Richmond R. C. Woolf B. A. R. Davies N. M. Swanson S. A. VanderWeele T. J. (2021). Strengthening the Reporting of Observational Studies in Epidemiology Using Mendelian Randomisation (STROBE-MR): Explanation and Elaboration. Bmj 375, n2233. 10.1136/bmj.n2233 34702754 PMC8546498

[B36] Sommer A. Tielsch J. M. Katz J. Quigley H. A. Gottsch J. D. Javitt J. (19911960). Relationship Between Intraocular Pressure and Primary Open Angle Glaucoma Among White and Black Americans. Arch. Ophthalmol. 109 (8), 1090–1095. 10.1001/archopht.1991.01080080050026 1867550

[B37] Sommer I. Teufer B. Szelag M. Nussbaumer-Streit B. Titscher V. Klerings I. (2020). The Performance of Anthropometric Tools to Determine Obesity: a Systematic Review and Meta-Analysis. Sci. Rep. 10 (1), 12699. 10.1038/s41598-020-69498-7 32728050 PMC7391719

[B38] Song X.-Y. Puyang Z. Chen A.-H. Zhao J. Li X.-J. Chen Y.-Y. (2018). Diffusion Tensor Imaging Detects Microstructural Differences of Visual Pathway in Patients with Primary Open-Angle Glaucoma and Ocular Hypertension. Front. Hum. Neurosci. 12, 426. 10.3389/fnhum.2018.00426 30459581 PMC6232882

[B39] Talari K. Goyal M. (2020). Retrospective Studies - Utility and Caveats. J. R. Coll. Physicians Edinb. 50 (4), 398–402. 10.4997/JRCPE.2020.409 33469615

[B40] Whitton C. Ho J. Tay Z. Rebello S. Lu Y. Ong C. (2017). Relative Validity and Reproducibility of a Food Frequency Questionnaire for Assessing Dietary Intakes in a Multi-Ethnic Asian Population Using 24-h Dietary Recalls and Biomarkers. Nutrients 9 (10), 1059. 10.3390/nu9101059 PMC5691676 28946670

[B41] Wise L. A. Rosenberg L. Radin R. G. Mattox C. Yang E. B. Palmer J. R. (2011). A Prospective Study of Diabetes, Lifestyle Factors, and Glaucoma Among African-American Women. Ann. Epidemiol. 21 (6), 430–439. 10.1016/j.annepidem.2011.03.006 21549278 PMC3091261

[B42] World Health Organization (2000). Obesity: Preventing and Managing the Global Epidemic. Report of a WHO Consultation. Technical Report Series, 894, i–xii. Geneva, Switzerland: World Health Organization, 1–253. 11234459

[B43] Wostyn P. De Groot V. Van Dam D. Audenaert K. Killer H. E. De Deyn P. P. (2017). Alzheimer's Disease and Glaucoma: Can Glymphatic System Dysfunction Underlie Their Comorbidity? Acta Ophthalmol. 95 (3), e244–e245. 10.1111/aos.13068 27126509

[B44] Wu F. Huang Y. Hu J. Shao Z. (2020). Mendelian Randomization Study of Telomere Length and Bone Mineral Density. Aging 13 (2), 2015–2030. 10.18632/aging.202197 33323545 PMC7880394

[B45] Wu Y. Y. Lye S. Briollais L. (2017). The Role of Early Life Growth Development, the FTO Gene and Exclusive Breastfeeding on Child BMI Trajectories. Int. J. Epidemiol. 46 (5), 1512–1522. 10.1093/ije/dyx081 29040503 PMC5837350

[B46] Yeung C. H. C. Schooling C. M. (2021). Systemic Inflammatory Regulators and Risk of Alzheimer's Disease: A Bidirectional Mendelian-Randomization Study. Int. J. Epidemiol. 50 (3), 829–840. 10.1093/ije/dyaa241 33313759

[B47] Yuan Y. Call M. K. Yuan Y. Zhang Y. Fischesser K. Liu C.-Y. (2013). Dexamethasone Induces Cross-Linked Actin Networks in Trabecular Meshwork Cells through Noncanonical Wnt Signaling. Invest. Ophthalmol. Vis. Sci. 54 (10), 6502–6509. 10.1167/iovs.13-12447 23963164 PMC3790389

[B48] Zarei R. Anvari P. Eslami Y. Fakhraie G. Mohammadi M. Jamali A. (2017). Retinal Nerve Fibre Layer Thickness Is Reduced in Metabolic Syndrome. Diabet. Med. 34 (8), 1061–1066. 10.1111/dme.13369 28430372

[B49] Zhang K. Dong S.-S. Guo Y. Tang S.-H. Wu H. Yao S. (2021). Causal Associations Between Blood Lipids and COVID-19 Risk: A Two-Sample Mendelian Randomization Study. Atvb 41, 2802–2810. 10.1161/atvbaha.121.316324 PMC8545250 34496635

