# Supplementary material for: The Causal Association Between Obesity and Primary Open-Angle Glaucoma: A Two-Sample Mendelian Randomization Study
Source: Front Genet. 2022 Apr 25;13:835524. doi: 10.3389/fgene.2022.835524 (PMC9081767; doi:10.3389/fgene.2022.835524)
Supplement: Supplementary file 1 [file DataSheet1.docx]

**Supplementary File**

Table 1. The single nucleotide polymorphism selected for BMI to perform Mendelian randomization analysis.

| **SNP** | **chr** | **EA_**  **exposure** | **EA_**  **outcome** | **β_**  **exposure** | **β_**  **outcome** | **EAF**  **_outcome** | **se_**  **outcome** | ***p*_**  **outcome** | **se_**  **exposure** | ***F-*statistics** | ***p*_**  **exposure** |
| --- | --- | --- | --- | --- | --- | --- | --- | --- | --- | --- | --- |
| rs10132280 | 14 | A | A | -0.0221 | -0.0454 | 0.35030 | 0.0367 | 0.2167000 | 0.0033 | 44.84940 | 1.40088e-11 |
| rs1016287 | 2 | C | C | -0.0228 | -0.0121 | 0.74080 | 0.0398 | 0.7605990 | 0.0033 | 47.73554 | 4.35512e-12 |
| rs11030104 | 11 | G | G | -0.0416 | 0.0092 | 0.16750 | 0.0468 | 0.8446000 | 0.0037 | 126.41052 | 6.65733e-30 |
| rs11727676 | 4 | C | C | -0.0365 | -0.0503 | 0.08604 | 0.0631 | 0.4247000 | 0.0063 | 33.56639 | 6.24698e-09 |
| rs12446632 | 16 | A | A | -0.0399 | 0.0315 | 0.12520 | 0.0526 | 0.5499000 | 0.0044 | 82.23192 | 1.80884e-19 |
| rs13021737 | 2 | G | G | 0.0604 | -0.0226 | 0.83870 | 0.0476 | 0.6351000 | 0.0039 | 239.85273 | 5.43876e-54 |
| rs13078960 | 3 | G | G | 0.0290 | -0.0106 | 0.17210 | 0.0465 | 0.8193000 | 0.0038 | 58.24100 | 1.42298e-14 |
| rs13130484 | 4 | T | T | 0.0398 | 0.0280 | 0.47580 | 0.0350 | 0.4230000 | 0.0030 | 176.00444 | 8.01124e-41 |
| rs1421085 | 16 | C | C | 0.0803 | 0.0793 | 0.41500 | 0.0355 | 0.0253303 | 0.0030 | 716.45444 | 2.16770e-158 |
| rs1460676 | 2 | C | C | 0.0209 | -0.0045 | 0.19910 | 0.0436 | 0.9169000 | 0.0038 | 30.25000 | 4.97794e-08 |
| rs1528435 | 2 | T | T | 0.0175 | 0.0361 | 0.64000 | 0.0365 | 0.3221000 | 0.0030 | 34.02778 | 4.77397e-09 |
| rs17001654 | 4 | G | G | 0.0304 | 0.0523 | 0.11440 | 0.0559 | 0.3501000 | 0.0052 | 34.17751 | 5.03095e-09 |
| rs17724992 | 19 | G | G | -0.0196 | -0.0655 | 0.22290 | 0.0420 | 0.1189000 | 0.0034 | 33.23183 | 7.78700e-09 |
| rs2112347 | 5 | G | G | -0.0254 | -0.0200 | 0.41520 | 0.0357 | 0.5756000 | 0.0030 | 52.82808 | 1.96020e-17 |
| rs2183825 | 9 | C | C | 0.0241 | -0.0349 | 0.37640 | 0.0362 | 0.3354000 | 0.0032 | 56.71973 | 2.22280e-14 |
| rs2820292 | 1 | C | C | 0.0181 | -0.0287 | 0.55260 | 0.0350 | 0.4116000 | 0.0029 | 38.95482 | 5.45205e-10 |
| rs2836754 | 21 | C | C | 0.0169 | 0.0348 | 0.47180 | 0.0352 | 0.3226000 | 0.0030 | 31.73444 | 1.60498e-08 |
| rs3736485 | 15 | G | G | -0.0160 | -0.0251 | 0.58670 | 0.0355 | 0.4802000 | 0.0029 | 30.43995 | 4.52397e-08 |
| rs3800229 | 6 | T | T | 0.0175 | 0.0414 | 0.63710 | 0.0364 | 0.2545000 | 0.0032 | 29.90723 | 4.95005e-08 |
| rs3888190 | 16 | A | A | 0.0311 | -0.0172 | 0.41350 | 0.0354 | 0.6283000 | 0.0030 | 107.46778 | 3.45382e-25 |
| rs4130548 | 1 | C | C | 0.0205 | -0.0005 | 0.33600 | 0.0371 | 0.9897000 | 0.0031 | 43.73049 | 2.44794e-11 |
| rs543874 | 1 | G | G | 0.0497 | -0.0157 | 0.17620 | 0.0461 | 0.7327000 | 0.0037 | 180.43024 | 2.28718e-40 |
| rs657452 | 1 | G | G | -0.0227 | -0.0184 | 0.57360 | 0.0353 | 0.6021010 | 0.0031 | 53.62019 | 2.12324e-13 |
| rs6713510 | 2 | A | A | 0.0164 | -0.0001 | 0.56260 | 0.0350 | 0.9982000 | 0.0029 | 31.98098 | 1.97401e-08 |
| rs7138803 | 12 | A | A | 0.0320 | 0.0439 | 0.38150 | 0.0362 | 0.2246000 | 0.0030 | 113.77778 | 5.11446e-26 |
| rs7144011 | 14 | T | T | 0.0274 | 0.0750 | 0.23850 | 0.0409 | 0.0669098 | 0.0035 | 61.28653 | 6.04505e-15 |
| rs7591633 | 2 | G | G | -0.0163 | 0.0245 | 0.63960 | 0.0366 | 0.5025000 | 0.0030 | 29.52111 | 4.20601e-08 |
| rs7599312 | 2 | A | A | -0.0214 | 0.0188 | 0.3021 | 0.0383 | 0.62400100 | 0.0033 | 42.05326 | 4.73042e-11 |
| rs9374842 | 6 | T | T | 0.0196 | 0.0211 | 0.7260 | 0.0390 | 0.58789900 | 0.0034 | 33.23183 | 7.19797e-09 |
| rs943005 | 6 | C | T | 0.0444 | 0.1311 | 0.1996 | 0.0436 | 0.00264101 | 0.0038 | 136.52078 | 4.52376e-31 |
| rs9540493 | 13 | A | G | -0.0182 | -0.0164 | 0.4846 | 0.0352 | 0.64050000 | 0.0031 | 34.46826 | 3.95203e-09 |
|  |  |  |  |  |  |  |  |  |  |  |  |

BMI, body mass index; SNP, single nucleotide polymorphism; EA, effect allele; NEA, non effect allele; EAF, frequency of the effect allele from the corresponding study; β, the effect of the effect allele; se, the standard error of the beta; *p*, *P*-value from the GWAS.

Table 2. The single nucleotide polymorphism selected for waist circumference to perform Mendelian randomization analysis.

| **SNP** | **chr** | **EA_**  **exposure** | **EA_**  **outcome** | **β_**  **exposure** | **β_**  **outcome** | **EAF**  **_outcome** | **se_**  **outcome** | ***p*_**  **outcome** | **se_**  **exposure** | ***F-*statistics** | ***p*_**  **exposure** |
| --- | --- | --- | --- | --- | --- | --- | --- | --- | --- | --- | --- |
| rs10041657 | 5 | A | A | 0.025 | -0.0213 | 0.190800 | 0.0444 | 0.63140100 | 0.0040 | 39.0625 | 2.90001e-10 |
| rs11205277 | 1 | G | G | 0.027 | -0.0718 | 0.341200 | 0.0376 | 0.05633000 | 0.0036 | 56.25 | 1.29987e-13 |
| rs12493901 | 3 | A | A | -0.021 | 0.0393 | 0.488400 | 0.0350 | 0.26190000 | 0.0034 | 38.14879 | 8.30004e-10 |
| rs12679556 | 8 | G | G | 0.026 | 0.0319 | 0.246500 | 0.0407 | 0.43390000 | 0.0039 | 44.44444 | 1.29987e-11 |
| rs13210323 | 6 | C | C | -0.022 | 0.0538 | 0.202800 | 0.0435 | 0.21570000 | 0.0038 | 33.51801 | 1.40001e-08 |
| rs16957304 | 16 | G | G | -0.059 | 0.0393 | 0.039510 | 0.0883 | 0.65600000 | 0.0110 | 28.7686 | 2.50000e-08 |
| rs2047937 | 16 | T | T | -0.019 | 0.0384 | 0.433200 | 0.0353 | 0.27740000 | 0.0034 | 31.22837 | 4.70002e-08 |
| rs2052670 | 2 | G | G | 0.020 | -0.0181 | 0.368200 | 0.0362 | 0.61750000 | 0.0035 | 32.65306 | 1.50000e-08 |
| rs2160077 | 14 | A | A | -0.018 | -0.0476 | 0.397600 | 0.0357 | 0.18200000 | 0.0033 | 29.75207 | 4.49997e-08 |
| rs2179129 | 22 | G | G | -0.019 | -0.0386 | 0.377800 | 0.0362 | 0.28600000 | 0.0034 | 31.22837 | 2.59998e-08 |
| rs2214442 | 7 | G | G | 0.026 | -0.0230 | 0.411100 | 0.0354 | 0.51590100 | 0.0045 | 33.38272 | 3.89996e-09 |
| rs2638953 | 12 | C | C | 0.024 | 0.0874 | 0.695000 | 0.0379 | 0.02125980 | 0.0036 | 44.44444 | 6.49980e-11 |
| rs272869 | 5 | G | G | 0.021 | -0.0093 | 0.507100 | 0.0350 | 0.79160000 | 0.0034 | 38.14879 | 6.69993e-10 |
| rs3786897 | 19 | G | G | 0.020 | 0.0491 | 0.411700 | 0.0357 | 0.16820000 | 0.0035 | 32.65306 | 8.79995e-09 |
| rs3791679 | 2 | G | G | -0.035 | 0.0708 | 0.228100 | 0.0416 | 0.08893030 | 0.0039 | 80.53912 | 2.09991e-19 |
| rs395962 | 6 | G | G | -0.029 | -0.0382 | 0.682300 | 0.0376 | 0.30900000 | 0.0036 | 64.89198 | 1.29987e-15 |
| rs4239436 | 18 | G | G | 0.040 | 0.0424 | 0.775200 | 0.0419 | 0.31130000 | 0.0041 | 95.18144 | 1.00000e-22 |
| rs459193 | 5 | G | G | -0.025 | -0.0429 | 0.999899 | 2.2667 | 0.98490000 | 0.0038 | 43.28255 | 7.70016e-11 |
| rs7162542 | 15 | G | G | 0.038 | 0.0348 | 0.549600 | 0.0350 | 0.32050000 | 0.0034 | 124.9135 | 9.70063e-29 |
| rs7621331 | 3 | G | G | -0.021 | 0.0041 | 0.299400 | 0.0381 | 0.91510000 | 0.0036 | 34.02778 | 9.40005e-09 |
| rs7684221 | 4 | A | A | -0.026 | 0.0431 | 0.092350 | 0.0598 | 0.47080000 | 0.0047 | 30.60208 | 4.20001e-08 |
| rs7801581 | 7 | T | T | 0.027 | -0.0247 | 0.243900 | 0.0408 | 0.54420100 | 0.0042 | 41.32653 | 8.00018e-11 |
| rs7970350 | 12 | T | T | -0.019 | 0.1084 | 0.476200 | 0.0351 | 0.00198399 | 0.0034 | 31.22837 | 3.79997e-08 |
| rs798489 | 7 | T | T | -0.025 | -0.0262 | 0.342300 | 0.0367 | 0.47390000 | 0.0037 | 45.65376 | 1.29987e-11 |
| rs806794 | 6 | G | G | -0.030 | 0.0271 | 0.416300 | 0.0359 | 0.45120000 | 0.0037 | 65.74142 | 1.90020e-15 |
| rs822531 | 7 | T | T | 0.024 | -0.0999 | 0.828200 | 0.0470 | 0.03361010 | 0.0044 | 29.75207 | 3.69999e-08 |
| rs849140 | 7 | C | C | -0.029 | 0.0914 | 0.607800 | 0.0359 | 0.01084000 | 0.0034 | 72.75087 | 4.70002e-17 |
| rs9389986 | 6 | A | A | -0.024 | 0.0017 | 0.2598 | 0.0400 | 0.9664000 | 0.0037 | 42.07451 | 5.70033e-11 |
| rs979012 | 20 | C | C | -0.033 | 0.0670 | 0.7061 | 0.0386 | 0.0824594 | 0.0036 | 84.02778 | 5.40008e-20 |
| rs984222 | 1 | G | G | 0.036 | 0.0171 | 0.7257 | 0.0391 | 0.6618000 | 0.0035 | 105.7959 | 1.50003e-25 |
| rs9864077 | 3 | C | C | -0.022 | -0.0044 | 0.2998 | 0.0382 | 0.9093000 | 0.0037 | 35.35427 | 1.29999e-09 |
| rs9977276 | 21 | G | G | 0.022 | -0.0229 | 0.7831 | 0.0425 | 0.5895000 | 0.0040 | 30.25 | 4.39997e-08 |
| rs998584 | 6 | A | A | 0.029 | -0.0093 | 0.4856 | 0.0352 | 0.7909990 | 0.0038 | 58.241 | 6.40030e-15 |
|  |  |  |  |  |  |  |  |  |  |  |  |

SNP, single nucleotide polymorphism; EA, effect allele; EAF, frequency of the effect allele from the corresponding study; β, the effect of the effect allele; se, the standard error of the beta; *p*, *P*-value from the GWAS.

Table 3. The single nucleotide polymorphism selected for hip circumference to perform Mendelian randomization analysis.

| **SNP** | **chr** | **EA_**  **exposure** | **EA_**  **outcome** | **β_**  **exposure** | **β_**  **outcome** | **EAF**  **_outcome** | **se_**  **outcome** | ***p*_**  **outcome** | **se_**  **exposure** | ***F-*statistics** | ***p*_**  **exposure** |
| --- | --- | --- | --- | --- | --- | --- | --- | --- | --- | --- | --- |
| rs10132280 | 14 | A | A | -0.022 | -0.0454 | 0.3503 | 0.0367 | 0.21670000 | 0.0038 | 33.51801 | 4.79999e-09 |
| rs10938397 | 4 | G | G | 0.030 | 0.0259 | 0.4768 | 0.0349 | 0.45880000 | 0.0037 | 65.74142 | 9.30037e-17 |
| rs11165623 | 1 | A | A | 0.022 | 0.0057 | 0.4221 | 0.0355 | 0.87320000 | 0.0035 | 39.5102 | 2.99999e-10 |
| rs12446632 | 16 | A | A | -0.036 | 0.0315 | 0.1252 | 0.0526 | 0.54990000 | 0.0052 | 47.92899 | 1.10002e-11 |
| rs13098327 | 3 | A | A | 0.027 | -0.0086 | 0.1712 | 0.0466 | 0.85420000 | 0.0044 | 37.65496 | 1.20000e-09 |
| rs13695 | 17 | T | T | 0.024 | 0.0094 | 0.2148 | 0.0426 | 0.82430000 | 0.0044 | 29.75207 | 4.30002e-08 |
| rs1548457 | 7 | C | C | 0.025 | 0.0682 | 0.3582 | 0.0362 | 0.05979020 | 0.0045 | 30.8642 | 1.70000e-08 |
| rs16894959 | 6 | C | C | 0.037 | -0.0457 | 0.2015 | 0.0446 | 0.30510000 | 0.0049 | 57.01791 | 9.60064e-14 |
| rs16905212 | 8 | C | C | -0.021 | -0.0139 | 0.3354 | 0.0370 | 0.70720000 | 0.0038 | 30.54017 | 1.89998e-08 |
| rs2112347 | 5 | G | G | -0.025 | -0.0200 | 0.4152 | 0.0357 | 0.57560000 | 0.0036 | 48.22531 | 9.70063e-12 |
| rs2206277 | 6 | T | T | 0.039 | 0.1270 | 0.2097 | 0.0429 | 0.00306098 | 0.0046 | 71.88091 | 3.10027e-17 |
| rs2293576 | 11 | A | A | -0.023 | -0.0292 | 0.2702 | 0.0401 | 0.46590000 | 0.0038 | 36.63435 | 2.10000e-09 |
| rs2820443 | 1 | C | C | 0.034 | 0.1056 | 0.2979 | 0.0383 | 0.00587205 | 0.0039 | 76.00263 | 4.90004e-18 |
| rs3800229 | 6 | T | T | 0.021 | 0.0414 | 0.6371 | 0.0364 | 0.25450000 | 0.0038 | 30.54017 | 3.20000e-08 |
| rs3888190 | 16 | A | A | 0.035 | -0.0172 | 0.4135 | 0.0354 | 0.62830000 | 0.0036 | 94.5216 | 9.39940e-22 |
| rs4132228 | 3 | T | T | 0.021 | -0.0261 | 0.2413 | 0.0409 | 0.52310000 | 0.0039 | 28.99408 | 3.09999e-08 |
| rs543874 | 1 | G | G | 0.045 | -0.0157 | 0.1762 | 0.0461 | 0.73270000 | 0.0045 | 100 | 1.80011e-23 |
| rs6265 | 11 | T | T | -0.034 | 0.0050 | 0.1558 | 0.0480 | 0.91660000 | 0.0045 | 57.08642 | 1.50003e-14 |
| rs6569648 | 6 | T | T | -0.029 | -0.0385 | 0.7879 | 0.0427 | 0.36650000 | 0.0042 | 47.67574 | 6.49980e-12 |
| rs6755502 | 2 | C | C | 0.054 | -0.0228 | 0.8388 | 0.0476 | 0.63219900 | 0.0047 | 132.0054 | 2.39994e-30 |
| rs7138803 | 12 | A | A | 0.029 | 0.0439 | 0.3815 | 0.0362 | 0.22460000 | 0.0037 | 61.4317 | 1.90020e-15 |
| rs7144011 | 14 | T | T | 0.030 | 0.0750 | 0.2385 | 0.0409 | 0.06690980 | 0.0042 | 51.02041 | 8.60003e-13 |
| rs806794 | 6 | G | G | -0.032 | 0.0271 | 0.4163 | 0.0359 | 0.45120000 | 0.0040 | 64 | 2.80027e-16 |
| rs9939973 | 16 | A | A | 0.070 | 0.0839 | 0.4246 | 0.0354 | 0.01773010 | 0.0036 | 378.0864 | 2.29985e-86 |
|  |  |  |  |  |  |  |  |  |  |  |  |

SNP, single nucleotide polymorphism; EA, effect allele; EAF, frequency of the effect allele from the corresponding study; β, the effect of the effect allele; se, the standard error of the beta; *p*, *P*-value from the GWAS.
